# Incongruence between genetic and morphological diversity in *Microcebus griseorufus *of Beza Mahafaly

**DOI:** 10.1186/1471-2148-6-98

**Published:** 2006-11-16

**Authors:** Kellie L Heckman, Emilienne Rasoazanabary, Erica Machlin, Laurie R Godfrey, Anne D Yoder

**Affiliations:** 1 Department of Ecology and Evolutionary Biology, Yale University, P.O. Box 208106, 165 Prospect St., New Haven, CT 06511 USA; 2 Department of Anthropology, 240 Hicks Way, University of Massachusetts, Amherst, MA 01003 USA; 3 Departments of Biology and Biological Anthropology & Anatomy, Duke University, Box 90338, Durham, NC 27708 USA

## Abstract

**Background:**

The past decade has seen a remarkable increase in the number of recognized mouse lemur species (genus *Microcebus*). As recently as 1994, only two species of mouse lemur were recognized according to the rules of zoological nomenclature. That number has now climbed to as many as fifteen proposed species. Indeed, increases in recognized species diversity have also characterized other nocturnal primates – galagos, sportive lemurs, and tarsiers. Presumably, the movement relates more to a previous lack of information than it does to any recent proclivity for taxonomic splitting. Due to their nocturnal habits, one can hypothesize that mouse lemurs will show only minimal variation in pelage coloration as such variation should be inconsequential for the purposes of mate and/or species recognition. Even so, current species descriptions for nocturnal strepsirrhines place a good deal of emphasis on relatively fine distinctions in pelage coloration.

**Results:**

Here, we report results from a multi-year study of mouse lemur populations from Beza Mahafaly in southern Madagascar. On the basis of morphological and pelage variation, we initially hypothesized the presence of up to three species of mouse lemurs occurring sympatrically at this locality, one of which appeared to be undescribed. Genetic analysis reveals definitively, however, that all three color morphs belong to a single recognized species, *Microcebus griseorufus*. Indeed, in some cases, the three color morphs can be characterized by identical mitochondrial haplotypes.

**Conclusion:**

Given these results, we conclude that investigators should always proceed with caution when using a single data source to identify novel species. A synthetic approach that combines morphological, genetic, geographic, and ecological data is most likely to reveal the true nature of species diversity.

## Background

A remarkable amount of primate diversity remains undocumented due to cryptic variation among species. To accurately and thoroughly document this diversity, genetic and/or behavioral investigations, in addition to morphological analyses, are necessary. The phenomenon of cryptic diversity is being actively explored, particularly for nocturnal primates [1-11]. Mouse lemurs (genus *Microcebus*) can potentially be said to represent a cryptic species radiation. They are the world's smallest living primates, with brown pelage and average adult body size ranging from 30 to 72 grams [12]. Given that they are strictly nocturnal, theory [13-15] would predict that mouse lemurs will emphasize olfactory and auditory communication signals over visual signals, as has been demonstrated for other nocturnal primates [1,2], [5,6], [16-18]. An array of studies conducted on mouse lemurs within the past several years appears to confirm this prediction. For example, exposure to female urine can significantly increase testosterone levels in males, just as exposure to the urine of dominant males can suppress testosterone production in other males [19]. Similarly, acoustic studies have revealed remarkable subtleties in signaling, with two noteworthy results that have direct implications for potential speciation mechanisms. Acoustic signals in mouse lemurs appear to evolve extremely rapidly, with the greatest levels of acoustic separation occurring in the sexual advertisement calls of males [11,20,21]. Thus, it is not surprising that morphological features might be only subtly variable in mouse lemurs, making them difficult to distinguish with human eyes. As with other cryptic species radiations, empirical recognition of species boundaries will depend on the reciprocal illumination obtained from a comparison of genetic and morphological data. The results of these analyses will then form working hypotheses of species limits, which can then be further tested in the field (e.g., testing for areas of non-interbreeding sympatry and/or variations in olfactory and/or acoustic signaling).

In 1972, Martin [22] recognized only two species of mouse lemur (up from one): *Microcebus murinus*, a small-eared, gray form, and *M. rufus*, a large-eared, reddish-brown form. This taxonomy was standard until the last decade of the twentieth century (see, for example, [23]). In the mid-1990s beginning with the work of Schmid and Kappeler, two additional species were added to the roster on the basis of variation in morphometric and coat color characteristics [24,25]. Then, in a geographically-broad morphological study that considered cranial, dental, and postcranial traits, Rasoloarison et al. [12] differentiated seven species of mouse lemurs from western Madagascar alone. These species were also described as identifiable by subtle differences in pelage coloration as well as dental and other morphological characteristics. Rasoloarison et al. [12] suggested that, by lumping "red" and "gray" forms into only two species, earlier researchers had underestimated the species diversity within the genus.

Molecular phylogenetic methods provide an alternative, powerful tool for examining the relationships and potential species boundaries of mouse lemurs [9,10], [26,27]. These methods identify species as genetic clades that may be comprised of individuals even from disparate geographic locations. This approach also provides the additional benefit of potentially identifying specimens of unknown origins, or of elucidating species identity by examining specimen positions on phylogenetic trees. This strategy was previously used to classify mouse lemur specimens collected in the Berenty Private Reserve (in southeastern Madagascar) from two forest types [10]. The resulting phylogenetic tree demonstrated that the study specimens grouped into two mouse lemur species clades, identifying a single individual as *M. murinus *and multiple individuals as *M. griseorufus*. Thus, two species of mouse lemur were identified as inhabiting the region of the Berenty Private Reserve. These two species exhibited microhabitat separation at Berenty: individuals identified as *M. griseorufus *were captured in the spiny forest, while the single individual captured in the gallery forest was determined to be *M. murinus*. As such, microhabitat separation of the two species at Berenty seemed evident, and concordant with the observation that *M. murinus *inhabits a lusher forest bordering a river at Kirindy, while *M. griseorufus *was known from drier forests at Beza Mahafaly.

**Figure 1:**
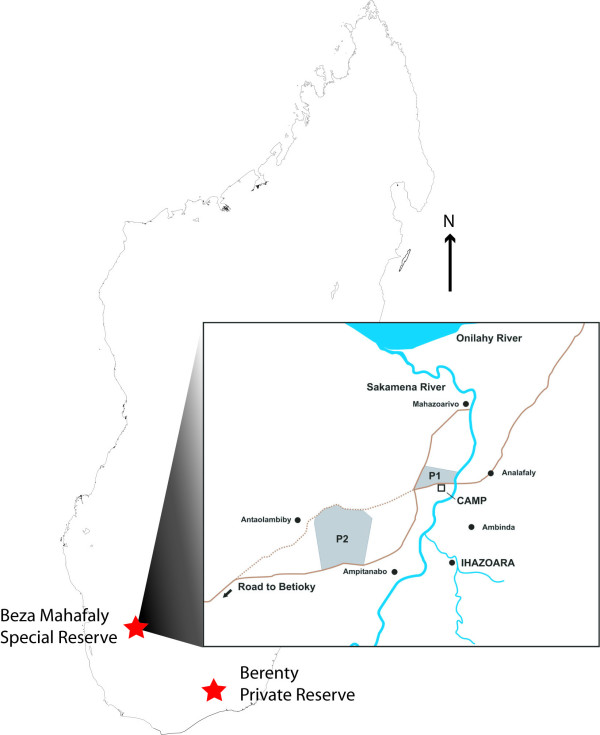
Map of the Beza Mahafaly region and sampling locations. P1 and P2 indicate the locations of the two parcels that belong to the reserve. The Ihazoara River is a tributary to the Sakamena River, which in turn flows into the Onilahy River to the north. The Ihazoara dry forest surrounds the village of Ihazoara. Locations of additional villages within a radius of 7 km from the reserve are also shown. For scale, the distance across P1 (east to west) is 1.25 km.

We used a similar approach to classify wild-caught mouse lemur individuals from Beza Mahafaly, a Special Reserve in southwestern Madagascar composed of two disparately sized, noncontiguous parcels separated by several kilometers. We captured mouse lemurs at three locations (all within a radius of about 7 km), including the dry forest at Ihazoara, but also two locations within the Reserve proper – a gallery forest (Parcel 1) bordering the Sakamena River, and a spiny forest (Parcel 2) dominated by succulent vegetation, located further from the Sakamena River [28] (Figure 1). The dry forest at Ihazoara is intermediate in vegetation characteristics between that of the spiny and gallery forests, but more similar to the spiny forest.

Previous researchers had inferred the presence of *M. murinus *at Beza Mahafaly [23,29,30], but no one had actually studied them in this region until Goodman [31,32] collected osteological specimens from owl pellets outside the reserve, and found them to contain large numbers of jaws and postcranial bones of mouse lemurs. Rasoloarison et al. [12] identified all but one of the jaws as belonging to *M. griseorufus*; the outlier appeared to be *M. murinus*. Additionally, six mouse lemurs captured by Rasoloarison at Ihazoara were all identified as *M. griseorufus *[12].

**Figure 2:**
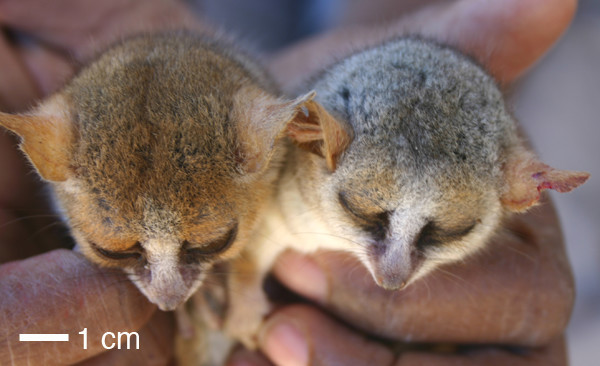
Two morphotypes collected at Beza Mahafaly. On the left is 0659-D2FC (the "all-red" variant), and on the right is 0659-CE82 (with "typical" *M. griseorufus *coloration). Both of these individuals were found in the gallery forest. Photo by L.R. Godfrey.

Rasoazanabary [28] began a long-term program of intensive monitoring of mouse lemurs at Beza Mahafaly in 2003, and individuals captured and released in 2003 for this behavioral study are the subjects of the molecular phylogenetic analysis presented herein. Most interesting was her discovery in 2003 of individuals of differing pelage coloration. The majority of captured individuals shared the "typical" *M. griseorufus *pattern, consisting of a red-brown tail, shades of gray and brown on the back, a red-brown stripe of varying intensity along the dorsal midline, white underside, white stripe between the eyes, and reddish-brown markings above the eyes, converging in an apex (the "reversed V") on an otherwise gray cap. However, six individuals, all captured in the spiny forest, resembled more strongly the pattern typical of *M. murinus *in the region of Kirindy (red-brown tail, gray back lacking a dorsal midline stripe, cream underside, and no facial markings or reverse V on the cap). Moreover, two individuals (one in the gallery forest and one in the spiny forest) had a unique appearance (red-brown tail, red-brown back lacking a dorsal midline stripe, cream underside, red-brown face and cap but with a creamy white stripe between the eyes) (see Figure 2).

Rasoazanabary conducted additional captures and focal individual follows of mouse lemurs in the three forest habitats at Beza Mahafaly during the years 2004 and 2005 (for a cumulative total, with 2003, of 14 months). About 17% (i.e., 15) of the individuals captured in 2003 were recaptured in 2004, 2005, or both. In all, 196 individuals were captured and marked during the 14-month sampling period. Of these, 13 (about 7%) showed *murinus*-like coloration, 165 (about 84%) showed typical *griseorufus *coloration, and 18 (about 9%) showed the "all-red" coloration.

The objective of the present study is to use molecular phylogenetic analysis to determine the placement of individuals of different pelage coloration within the larger mouse lemur phylogeny, and thus to investigate species identity using genetic evidence. Our *a priori *hypothesis was that individuals that displayed a *Microcebus murinus*-like coat coloration would fall into the *M. murinus *clade and *M. griseorufus*-like individuals into the *M*. *griseorufus *clade. We also hypothesized that the "all-red" individuals would form a novel clade in the *Microcebus *tree. In addition to examining the broader phylogenetic relationships, we employed molecular techniques to examine the relationships among individuals at the three forests, and thus to test whether geography has played a significant role in the generation of intraspecific variation. The genetic and morphological data were tested for structure with respect to three sampling locations in the Beza Mahafaly region. As the three sites are ecologically and geographically distinct (two located within the reserve and one outside, and on the opposite side of the Sakamena River), we aimed to determine whether the river and fields separating them, or the different microhabitats they represent, are potential barriers to gene flow.

## Results

We examined the relationships of seventy specimens from Beza Mahafaly, in addition to six samples of *M. griseorufus *previously obtained from Ihazoara and eleven samples of *M. griseorufus *from the Berenty Reserve. From the 70 cytochrome b sequences produced in this study, 44 haplotypes were found. Included in the analysis were DNA sequence data of the 70 individuals sampled from Beza Mahafaly, in addition to thirty-eight published sequences of *Microcebus*, representing seven recognized species (Table 1) and two species of dwarf lemurs (*Cheirogaleus major *and *Cheirogaleus medius*) from the same taxonomic family, Cheirogaleidae.

**Table 1:** Genbank accession numbers for sequences used in the molecular analyses

Species	Genbank No.	Reference
*Microcebus griseorufus*	AF285567–AF285568	9
	AY167065–AY167070; AY167072–AY167076	10
	DQ979888–DQ979958	de novo
*M. murinus*	AF285557–AF285566	9
	AY167071	10
	U53572	31
*M. berthae*	AF285540–AF285543	9
*M. myoxinus*	AF285536; AF285538	9
*M. ravelobensis*	AF285529–AF285532	9
*M. rufus*	AF285549; AF285551	9
*M. sambiranensis*	AF285554–AF285556	9
*M. tavaratra*	AF285533–AF285534	9
*Cheirogaleus major*	AY605911	34
*C. medius*	AY605909	34

**Figure 3:**
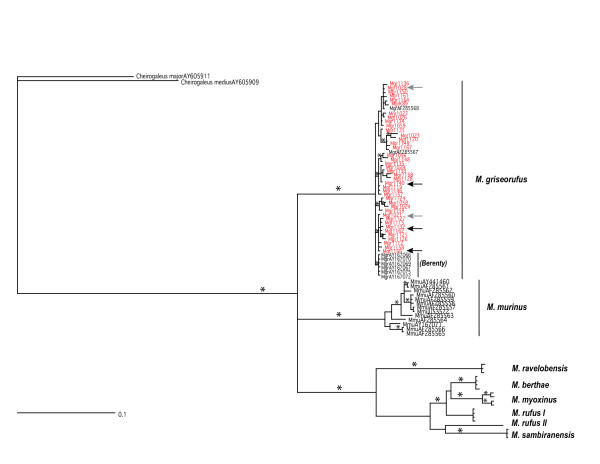
Phylogenetic tree of *Microcebus *derived from cytochrome b sequences. Asterisks along branches indicate posterior probabilities greaters than 95%. *Microcebus *sequences generated during the course of this study are in red. Gray arrows are indicative of haplotypes from individuals with the all-red variant. Black arrows are indicative of haplotypes from individuals with the *murinus*-type variant.

Phylogenetic analyses revealed that all Beza Mahafaly individuals collected for this study were clearly nested within a *Microcebus griseorufus *clade, composed of individuals from both the Beza Mahafaly and Berenty regions, regardless of coat color or sampling location within Beza Mahafaly (Figure 3). In addition, individuals that displayed murinus-like or unique pelage patterns shared a haplotype with individuals bearing the more common griseorufus form. The specimens from Berenty formed a distinct clade nested within the greater *M. griseorufus *clade.

We examined the *M. griseorufus *sequences for geographic structuring of haplotypes from the two collection sites, Beza Mahafaly and Berenty. The AMOVA revealed strong genetic structure separating individuals from Beza Mahafaly from individuals from Berenty (φ_st _= 0.3552). MIGRATE analyses consistently yielded the highest population size in Parcel 2 within the reserve, the spiny habitat (0.024); while Parcel 1, the gallery forest, had the smallest (0.0006; Ihazoara: 0.004). This result is consistent with the density patterns observed in the field. Migration rate analyses revealed that most *M. griseorufus *movement was leading into the spiny habitat from the other two populations, though gene flow was bidirectional among pair-wise combinations of all three locations.

**Figure 4:**
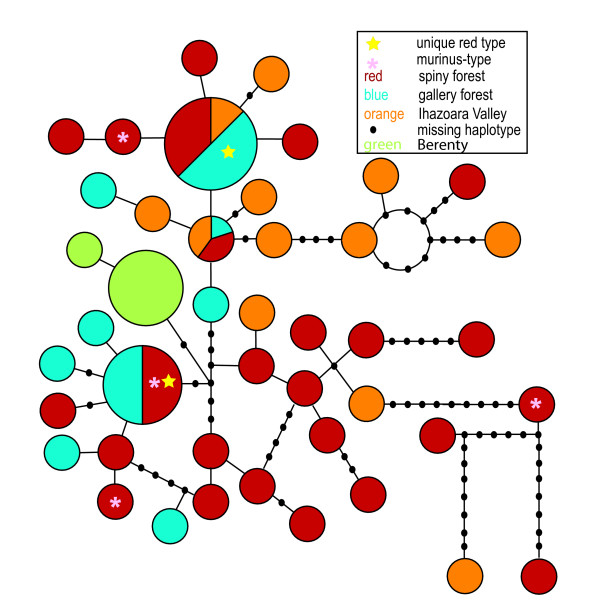
This is the network of Beza Mahafaly and Berenty cytochrome b haplotypes. In this figure each color represents a separate habitat type and each circle represents a single haplotype. The number of individuals that share each haplotype is drawn in proportion to the size of each circle.

The haplotype network (Figure 4) visually demonstrates the distribution of the atypical *M. griseorufus *morphotypes. These morphotypes share common cytochrome *b *haplotypes with individuals of typical coat coloration. Also, there are common haplotypes that are shared between multiple sampling sites. However, haplotypes that show greater divergence from the common haplotypes, five or more missing haplotypes connecting two haplotypes, are more often from the spiny forest (5 occurrences) or from Ihazoara (2 occurrences), compared to the gallery forest (no occurrences).

Chi-square tests of the differences in distributions of pelage types across the three forests fail to reveal significant differences (Table 2, 3, 4); instead, the distributions are remarkably similar in all three. However, discriminant function analysis shows significant morphometric differences between populations in the three forests. Only the first canonical function (with a Wilks' Lambda of .73 and a chi-square of 30.22, df = 12) is statistically significant (*p *= .003). This function accounts for 83.3% of the variance, and separates individuals from the gallery forest (with positive scores) from those in the spiny forest (with negative scores). There is considerable overlap of scores of individuals from each forest type, with individuals from Ihazoara intermediate and most likely to be classified as coming from one of the other sites. The post hoc classification success for all individuals is 55.3%, with 69.8% of individuals from the spiny forest, 50% of individuals from the gallery forest, and only 40% of individuals from Ihazoara correctly classified. The structure matrix, when considered in conjunction with the centroid scores of individuals from each of the three forests (Table 5), reveals that individuals from the spiny forest tend to have shorter skulls, ears, and bodies than individuals at either of the two other sites, but especially the gallery forest. Essentially, individuals from the spiny forest are smaller in body size than those from other forests. ANOVA confirms that these differences are statistically significant (Table 6), even at a univariate level. The three additional variables (tail length, bizygomatic breadth, and canine height) are poorly correlated with scores on this axis, and two (bizygomatic breadth and canine height) do not vary significantly by site.

**Table 2:** Pearson's chi-square tests of pelage differences by habitat: dorsal fur (Chi-square = .64, df = 4, p = .96, NS)

	Habitat			
		
Dorsal fur color	Gallery	Ihazoara	Spiny	Total
Grey	3	3	7	13
Grey-Brown	46	45	74	165
Red	4	5	9	18
Total	53	53	90	196

**Table 3:** Pearson's chi-square tests of pelage differences by habitat: reversed V (Chi-square = .001, df = 2, p = 1.00, NS)

	Habitat			
		
Presence of reversed "V"	Gallery	Ihazoara	Spiny	Total
Absent or indistinct	7	7	12	26
Distinct	46	46	78	170
Total	53	53	90	196

**Table 4:** Pearson's chi-square tests of pelage differences by habitat: dorsal median stripe (Chi-square = .84, df = 2, p = .66, NS)

	Habitat			
		
Appearance of dorsal stripe	Gallery	Ihazoara	Spiny	Total
Absent or indistinct	5	6	13	24
Distinct	48	47	77	172
Total	53	53	90	196

**Table 5:** Structure matrix, canonical discriminant function analysis of morphometric traits of mouse lemurs in the three habitats*

Trait	Correlation with Function 1
Skull length	.80
Ear length	.73
Body length	.67
Tail length	.45
Canine height	.29
Bizygomatic breadth	-.09

*Group centroid scores for Function 1: Gallery = .56, Ihazoara = .33, Spiny = -.62.

**Table 6:** Univariate ANOVAs for morphometric trait variation by habitat (Means in mm)

Trait (mm)	Gallery Mean (SD)	Ihazoara Mean (SD)	Spiny Mean (SD)	Total Mean (SD)	F	*P *value
Skull length	33.3 (.83)	32.6 (1.26)	31.4 (2.55)	32.3 (1.99)	9.37	<.001
Ear length	22.8 (.87)	22.8 (1.30)	21.9 (1.07)	22.4 (1.17)	8.04	<.01
Body length	93.6 (8.00)	88.4 (10.23)	82.7 (15.63)	87.5 (13.02)	7.09	<.01
Tail length	146.4 (8.45)	147.5 (7.33)	142.0 (12.05)	144.9 (10.08)	3.25	<.05
Canine height	1.7 (.13)	1.7 (.15)	1.63 (.17)	1.7 (.15)	1.91	NS
Bizygomatic	20.3 (1.1)	20.1 (.95)	20.8 (.61)	20.4 (6.23)	0.13	NS

## Discussion

The results of molecular phylogenetic analyses of cytochrome b mtDNA sequences fail to support our initial hypothesis that mouse lemurs collected at Beza Mahafaly with *murinus*-like or unique pelage characteristics are either *M. murinus *or a novel species. All individuals form a single clade with individuals previously classified as *M. griseorufus*. Therefore, we believe that all seventy individuals sequenced should be classified as *M. griseorufus. M. griseorufus *has significantly diverged from its sister species, *M. murinus*, with both species forming distinct clades with significant posterior probability (>95%). The mouse lemurs with divergent coat characteristics were included in the *M. griseorufus *clade, as they shared identical mtDNA haplotypes with individuals displaying the more typical *M. griseorufus *morphotype. The complex color patterns are independent of habitat type as confirmed by chi-square tests (Table 2, 3, 4); they are also uncorrelated with genetic distance, as suggested by the distribution of haplotypes in the network (Figure 4).

It is instructive to consider the relative importance of visual, auditory, and olfactory signals in *M. griseorufus *social communication, and how variation in pelage coloration is likely to be perceived. Like other mouse lemurs, *Microcebus griseorufus *are nocturnal, solitary foragers with a dispersed social system. Encounters among individuals at Beza Mahafaly are common (indeed, while foraging, two mouse lemurs may occupy a single tree), but rarely are individuals in physical contact while active. As in other mouse lemurs, audition and olfaction are critical to social signaling. For example, *Microcebus murinus *has been shown in captivity to display group-specific vocalization patterns [21], as have male *M. murinus *in neighboring demes during the breeding season [20]. In addition, wild *M. ravelobensis *were shown to regulate inter- and intragroup spatial distributions using olfactory and acoustic signals [35]. In this species, individuals use different acoustic signals when sleeping groups disperse at sunset as opposed to when they gather at sunrise. These acoustic calls were found to be specific to each social group [35]. Zimmermann et al. [11] have shown that *M. murinus *can be distinguished from *M. rufus *using vocal fingerprinting. Olfactory and auditory cues have not been studied in detail in *M. griseorufus*. Nevertheless, Rasoazanabary has observed the use of trill vocalizations to attract mates, and vocalizations can be heard during or just prior to agonistic encounters. Urine washing is common, and individuals have strong odors that are detectable even by human observers. Olfaction and audition are almost certain to be more important than vision in social encounters.

This is not to imply that vision is unimportant to mouse lemurs. Reproduction is photoperiod controlled, as is seasonal torpor [36-38]. Indeed, photoperiod appears to have an effect on life span in mouse lemurs [39]. On a daily basis, light intensity helps to regulate activity levels [40]; mouse lemurs do not emerge from their nests to forage until light levels are sufficiently low. Facial patterns (light and dark areas) may contribute to species or individual recognition [41]. As in almost all other strepsirrhines, mouse lemurs possess a tapetum lucidum to increase their sensitivity to low light intensity.

However, vision in mouse lemurs is dominated by rods (photoreceptor cells with high sensitivity to very low levels of illumination, and with a pigment showing maximum sensitivity to light in the green part of the spectrum) and is thus largely scotopic. This contrasts with primates that have photopic vision (dominated by cones, which are sensitive to varying light wavelengths, depending on pigment type). Cones are not active at low light levels, and rods have a restricted range of wavelength sensitivity, so vision may be expected to be achromatic for all strictly-nocturnal primates [42]. Furthermore, the density ratio of cones to rods is likely to be low in *M. griseorufus*. Dkhissi-Benyahya et al. [43] report a peak rod density of 850,000 rods/mm^2 ^and a peak cone density of 7,500 to 8,000 cones/mm^2 ^in *M. murinus*. Less than 0.2% of the cone population is represented by short wavelength-sensitive (SWS), as opposed to medium to long wavelength-sensitive, cells. Whereas *M. murinus *do possess a variety of cone types, their density ratio of cones to rods is very low, and SWS cones are irregularly distributed [43]. The irregular distribution and very low number of SWS cones preclude an important role for color vision, even at dusk or dawn [44]. In summary, the pelage color variation that is perceptible to humans is likely to be invisible to mouse lemurs.

Our genetic results demonstrate that coat coloration is not diagnostic of species differentiation at Beza Mahafaly. Indeed, pelage color variation may be problematic as an indicator of species boundaries for nocturnal primates in general. Why so much intraspecific variation in mouse lemur pelage coloration exists at Beza Mahafaly is unknown. In order to further investigate this phenomenon, we need more systematic data on the degree of coat color variation in populations of mouse lemurs in different geographic regions. We note that at Beza Mahafaly the three pelage types described here are not always discrete. Some individuals show combinations that can be considered intermediate between these types (e.g., gray dorsal fur with no brown fringe or highlights, but with a somewhat distinct dorsal stripe and reversed V).

With the molecular analysis, we determined that there is reciprocal gene flow among the three sampling sites within Beza Mahafaly. The lack of genetic structure and prevalence of dispersal between the parcels in the reserve and Ihazoara is noteworthy given our sampling of individuals on both sides of the Sakamena River, and in habitats separated today by other apparent barriers, such as cleared fields. Multiple studies have recently implicated rivers as important barriers to gene flow in lemur species [27,45,46]. Pastorini and colleagues [46] have determined that the Tsiribihina and Betsiboka Rivers in western and northwestern Madagascar, respectively, greatly hinder gene flow among species in the lemur genera *Eulemur*, *Propithecus*, *Lepilemur*, and possibly *Microcebus*. It is evident that the Sakamena River fails to do the same for mouse lemurs at Beza Mahafaly. However, the Betsiboka and Tsiribihina Rivers are far more formidable year-round than is the Sakamena and the even-smaller Ihazoara River. The Sakamena River is a tributary to the Onilahy River (to the north, more comparable to the Betsiboka or the Tsiribihina Rivers in size), and the Ihazoara is a much narrower tributary feeding into the Sakamena. The Sakamena and Ihazoara Rivers are dry for eight months every year, and the water is shallow even during the wettest months. Floating vegetation (following a cyclone) may occasionally provide pathways for mouse lemurs, allowing them to cross these narrow rivers, as anecdotal evidence suggests. Moreover, the distribution of forests in the region of Beza Mahafaly prior to the arrival of humans in the region over 2000 years ago [46] is not known. Our genetic data confirm that dispersal is occurring despite the separation of forests by potentially inhospitable space, and regardless of dispersal mechanism. This information is important if we are to construct and test hypotheses regarding dispersal mechanisms and determine the connectivity among forest fragments.

Whatever the mechanisms for geographic dispersal, it is clear that, at Beza Mahafaly, *M. griseorufus *is not limited to spiny-forest habitats, though dispersal patterns may indicate a preference. In contrast to the situation at Berenty where *M. griseorufus *has been described to occupy the spiny forest and *M. murinus *the gallery forest [10], *M. griseorufus *at Beza Mahafaly occupy gallery forests, dry forests, and spiny forests. How this species adapts to the very different microhabitats is the subject of the ongoing behavioral study at Beza Mahafaly by Rasoazanabary. Finally, it is apparent that, despite a lack of genetic structure of populations of mouse lemurs across the microhabitats at Beza Mahafaly, individuals from the spiny forest do differ slightly (but statistically significantly) from individuals in the gallery forest in such features as body length, ear length, and skull length. The developmental basis of this variation will also require further analysis. It is clear, however, that both habitats play an important role in the maintenance and possibly the development of diversity in this species and both should be a priority in future conservation efforts in this region.

## Conclusion

Using a combination of phylogenetics and population genetic methods, we were able to determine that all mouse lemur individuals sampled at Beza Mahafaly belong to the species *M. griseorufus*, regardless of pelage characteristics. Three pelage-color variants exist in all three forests, in roughly similar proportions. This evidence supports the hypothesis that non-visual cues are paramount in social interactions of individual mouse lemurs, and that, to the extent that vision is important, it does not depend on color discrimination. We also determined that mouse lemurs from ecologically distinct sampling locations display no genetic structuring.

While we are confident in the results produced in this study, it is limited as only a single mtDNA gene was used to make inferences. Therefore, we recommend that further work be performed to confirm the results and conclusions made in this study, primarily through the inclusion of nuclear genetic markers.

## Methods

### Field Methods

Between April 1 and August 15, 2003, 120 Sherman live traps were set at intervals of 25 m in 7.5-hectare sampling areas in each of the three forests. Sampling was conducted for a total of 23 days in each forest (69 days combined). A total of 89 *Microcebus *were captured (45 in the spiny forest, 21 in the gallery forest, and 23 at Ihazoara). Pieces of banana were used to lure mouse lemurs into the traps. Captured individuals were weighed using a Pesola spring scale and temporarily anesthetized (0.01 ml or less of telazol, depending on body mass). Anesthetized individuals were measured, marked, and released after full recovery from the effects of the anesthesia. Each individual was scored for the presence or absence of a reversed V, the presence or absence of a dorsal median stripe, the color of the fur, and the color of the tail. Skull length, bizygomatic breadth, body length, tail length, ear length and canine height were recorded for each captured individual. Clips (ca. 2 mm^2^) were taken from each ear, and preserved in 70% ethanol.

### Laboratory methods

Eighty ear tissue samples were delivered to Yale for molecular analysis; ten of these yielded no DNA or DNA of insufficient quality for analysis. Each had identifying field (or microchip) codes, but, to ensure blind analysis of the DNA, no information regarding location or pelage coloration accompanied the samples. Mitochondrial DNA was extracted using a QIAamp DNA Mini Kit (QIAGEN cat. no. 51306). The full cytochrome *b *gene region was amplified and sequenced for seventy of the sampled individuals using two pairs of primers L14724/H15261 and L15171/UMMZ [9]. The PCR protocol was 5:00 min. of 95.0° followed by 35 cycles of 95.0° for 0:45 sec., 52.0° for 0:45 sec., 72.0° for 1:00 min., and a final extension of 72.0° for 5:00 min. PCR products were cleaned with a QIAquick PCR purification kit (QIAGEN cat. no. 28106). The cleaned products were cycle sequenced using a big dye-terminator sequencing kit (Applied Biosystems, Foster City, CA). The sequences were analyzed by capillary electrophoresis with an Applied Biosystems Prizm 3100 genetic analyzer. Cytochrome *b *sequences were aligned by eye in Sequencher and exported into MacClade [47] for further editing.

### Molecular Analysis

Phylogenetic analysis of the molecular data was performed using Bayesian methods, implemented in Mr. Bayes v. 3.1.2 [48] using the model GTR+I+G. The model of evolution was selected with Modeltest v. 3.06 [49] and chosen based on the Akaike information criterion [50]. Identical haplotypes were represented only once in the analyzed phylogenetic dataset. Four Metropolis-coupled MCMC chains were run for ten million generations with trees sampled every 1000 generations. Tracer software 1.2 [51] was used to examine stationarity of log posteriors to estimate a burn, which was discarded.

An AMOVA [52] was performed in Arlequin v. 2.000 [53] to explore hierarchical patterns of population genetic structure between *M. griseorufus *at Beza Mahafaly and Berenty. AMOVA uses the frequencies of haplotypes and the number of mutations between them to test the significance of the variance components associated with various hierarchical levels of genetic structure (within populations, among populations within groups, and among groups) by means of non-parametric permutation methods [52]. Sampling sites were treated as individual populations to test for overall genetic subdivision. Uncorrected pair-wise distances were used to estimate the relative contribution of molecular variance of *M. griseorufus *at Beza Mahafaly and Berenty.

The program MIGRATE v.2.1.3 [54,55], was used to jointly estimate effective population sizes (Θ = N_e_*mu) and asymmetric dispersal rates (M = m/mu) between the three populations of *M. griseorufus *found in the Beza Mahafaly area. Parameters were estimated using the Bayesian search strategy [56], using default priors. MIGRATE runs were replicated to verify consistency, each replicate consisting of 10 short chains and four long chains that were heated (1.0, 2.3, 3.6, 9.0) for 10,000,000 steps excluding 10,000 steps as burn in.

A haplotype network was created using the software package TCS v. 1.21 [57]. The program collapses DNA sequences into haplotypes and calculates the frequencies of haplotypes in the sample. It then calculates an absolute distance matrix from which it estimates phylogenetic networks using a probability of parsimony, until the probability exceeds 0.95 [58].

### Analysis of morphological variation

We used SPSS Version 14 for our analysis of coat and morphometric characteristics. Pearson chi-square was used to determine the significance of differences in pelage coloration at the three sites. Discriminant function analysis of morphometric variables (body length, tail length, skull length, bizygomatic breadth, ear length, and canine height) was used to determine whether populations at the gallery forest, spiny forest, and Ihazoara could be distinguished from one another on the basis of a set of morphometric variables collected over the entire three-year period. Only first captures (196 individuals) were entered into these analyses, to avoid repeated sampling of the same individuals. Following Hoaglin and others [59], univariate analyses (ANOVA) were applied in an exploratory sense to determine the magnitude and direction of site differences for those variables found (using Discriminant Function Analysis) to distinguish mouse lemurs at the three sites.

## Authors' contributions

KLH designed the molecular portion of the study, carried out the molecular work, sequence data manipulation, and phylogenetic and populatation genetic analyses, and drafted the manuscript. ER designed the morphological portion of the study, collected field data, morphometric data, and conducted morphometric analyses. EM conducted the work generating DNA sequences. LG aided in the design of the morphological portion of the study, conducted morphometric analyses, and contributed to the manuscript draft. ADY aided in the design of the molecular work. All authors read and approved the final manuscript.
